# State-dependent responses of emotional brain networks in high myopia under blurred vision: a shift towards dorsal stream and cross-modal processing

**DOI:** 10.3389/fnhum.2026.1927265

**Published:** 2026-10-21

**Authors:** Lin Cui, Xianqing Zeng, Shuai Chang, Xinping Yu, Ming Meng

**Affiliations:** 1 Taizhou Vocational College of Science & Technology, Taizhou, China; 2 School of Educational Sciences, Lingnan Normal University, Zhanjiang, Guangdong, China; 3 Guangdong Key Laboratory of Mental Health and Cognitive Science, School of Psychology, South China Normal University, Guangzhou, China; 4 State Key Laboratory of Ophthalmology, Zhongshan Ophthalmic Center, Sun Yat-Sen University, Guangdong Provincial Key Laboratory of Ophthalmology and Visual Science, Guangdong Provincial Clinical Research Center for Ocular Diseases, Guangzhou, China

**Keywords:** cross-modal compensation, dorsal visual stream, emotional processing, functional connectivity, high myopia (HM)

## Abstract

**Purpose:**

Reliable sensory input plays a vital role in shaping emotional experience, however, how the brain modulates the formation of emotion under chronic visual degradation remains unclear. Using high myopia (HM) as a natural model of sustained blur, this study investigated state-dependent responses of emotional brain networks during blurred movie viewing.

**Methods:**

Twenty-four participants with high myopia (HM) were tested without optical correction, comparing with normal vision (NV) controls from previous research. All participants watched thriller and scenic movie clips twice over one week during fMRI. Intra-subject synchrony and seed-based functional connectivity analyses focused on the amygdala and visual processing streams.

**Results:**

Compared to NV controls, the HM group exhibited reduced intra-subject synchrony in visual cortices but increased synchrony in the primary auditory cortex, indicating cross-modal compensatory enhancement. During threat processing, HM participants showed strengthened and stable functional connectivity between the amygdala and the dorsal visual stream. For non-threatening content, this enhanced connectivity initially involved both dorsal and ventral stream but persisted selectively in the dorsal stream upon repeated viewing.

**Conclusion:**

Sustained visual blur elicits a perceptual-state-dependent functional response in emotional networks, characterized by heightened cross-modal auditory integration and an increased recruitment of the visual dorsal stream during blurred viewing. These findings highlight the brain’s immediate compensatory mechanisms under conditions of ambiguous visual input.

## Highlight

What are the main findings?

- Visual blur in high myopia is associated with cross-modal auditory enhancement, as evidenced by increased intra-subject synchrony in the primary auditory cortex during emotional movie viewing.
- High myopia individuals viewing blurred stimuli exhibit strengthened functional connectivity between the amygdala and the dorsal visual stream during threat processing, indicating a state-dependent shift in processing strategy.

What are the implications of the main findings?

- The canonical dissociation between consensual and idiosyncratic neural responses is attenuated under blurred viewing conditions, revealing a more unified, top-down dependent mode of emotional processing during acute sensory degradation.

## 1 Introduction

A fundamental pursuit in affective neuroscience is to understand how the brain constructs emotional experience. Combining neuroimaging and naturalistic paradigms, particularly movie viewing, has proven fruitful in this endeavor, capturing robust, ecologically valid brain responses and revealing significant inter-subject synchrony (ISC)—a key marker of shared, consensual experience across individuals (Hasson et al., 2004; Sonkusare et al., 2019; Zeng et al., 2025; Zhou et al., 2022). Central to this process is the amygdala, extensively documented for its role in initiating rapid, automatic, and conserved responses to fear-relevant stimuli (LaBar et al., 1998; LeDoux, 2007; Morris et al., 1999). Our previous work (Cui et al., 2021) reinforced this model, demonstrating that in NV individuals, repeated viewing of a horror movie elicits a stable, amygdala-centric consensual response, dissociable from more idiosyncratic, experience-driven neural patterns.

This canonical model, however, is established based on a critical yet implicit assumption: optimal sensory input. Previous studies have demonstrated that the quality and spatial-frequency composition of visual input can modulate emotional perception and associated neural responses (Guo et al., 2019; Vuilleumier et al., 2003). Consequently, the current understanding is derived from, and limited to, studies of neurotypical brains with normal-vision. A pivotal question remains: how is emotional experience constructed and regulated when the sensory input is chronically degraded? While external manipulation of stimulus clarity (e.g., blurring) can probe this transiently, it fails to capture the sustained neuroadaptive changes that may arise from the long-term development of adapting to perceptually ambiguous vision (Boucart et al., 2008; De Boer et al., 2021).

High myopia (HM) offers a unique and powerful “natural model” to bridge this gap. Moving beyond the traditional clinical focus on ocular pathology, a growing body of neuroimaging research confirms that HM fundamentally alters brain organization (Cheng et al., 2020). Studies have revealed widespread reductions in functional connectivity density, aberrant spontaneous activity in visual cortices, and impaired functional integration within the visual hierarchy (Hu et al., 2018; Ji et al., 2023; Zhai et al., 2016). Crucially, these findings suggest that the HM visual system is not merely degraded but exhibits altered functional responses to its compromised visual input.

This response pattern likely reflects an increased reliance on the dorsal visual stream (Mahon et al., 2013; Merigan and Maunsell, 1993). The primate visual system is well-known to be organized into a ventral stream (the ‘what’ stream), specializing in detailed object recognition using high spatial frequencies, and a dorsal stream (the ‘where/how’ stream), dedicated to processing spatial information, motion, and guiding action—functions that rely more on low spatial frequencies and are thus less susceptible to blur (Goodale and Milner, 1992; Livingstone and Hubel, 1988; Mishkin et al., 1983). We posit that chronic visual degradation disproportionately handicaps the ventral stream, forcing a strategic bias towards the dorsal stream to efficiently extract salient information from a noisy visual environment.

Building on the observation of neural responses under visual degradation and the potential utility of the dorsal stream, we propose that the emotional brain network of HM individuals viewing blurred stimuli deviates from the classic, stimulus-driven automaticity model. We hypothesize three state-dependent mechanisms that may reflect compensatory responses to acute visual blur:

(H1) Cross-Modal Compensation: To resolve perceptual uncertainty, HM individuals will exhibit enhanced neural synchrony and strengthened functional connectivity of the auditory cortex, reflecting a greater reliance on the intact auditory channel. (H2) Dorsal Stream Bias for Threat Processing: When evaluating potential threat, HM individuals will show preferential recruitment of the dorsal visual stream, evidenced by enhanced functional connectivity between the amygdala and dorsal stream regions (e.g., posterior parietal cortex), facilitating rapid, coarse-grained assessment. (H3) Attenuation of Consensual Processing Under Blur: When visual input is degraded, the brain may increase reliance on top-down, subjective experience to disambiguate blurred stimuli, potentially leading to a reduction in the clear dissociation between consensual and idiosyncratic neural networks observed in NV individuals (Cui et al., 2021). Thus, we predict overall lower intra-subject correlation and a reduced experience-dependent separation of amygdala networks under blurred viewing conditions.

By testing these hypotheses, our study aims to transcend the traditional clinical narrative of HM. We seek to uncover the unique neurocognitive strategies employed by the HM brain, offering a novel lens through which to understand how the brain responds under persistent perceptual challenge.

## 2 Materials and methods

### 2.1 Participants

Twenty-four volunteers with HM (12 female; mean age = 22.8 years) participated in the present fMRI experiment. The NV control data (*n* = 15; 9 female; mean age = 22.6 years) were obtained from our previous study conducted in 2020 (Cui et al., 2021), using the same MRI scanner, acquisition parameters, and stimulus paradigm. Participants with high myopia, defined as a spherical equivalent refractive error of −6.00 diopters or worse (≤ − 6.00 D) in both eyes (Tailor et al., 2024), were right-handed, and had normal hearing. None had a history of neurological or psychiatric disorders. The Institutional Review Board of South China Normal University approved the study. All participants provided written informed consent. Crucially, to ensure that the visual stimuli they received were subjectively blurred, all participants were scanned without optical correction (i.e., without wearing glasses or contact lenses). All HM participants were required to have a spherical equivalent refractive error of ≤ − 6.00 D in both eyes, confirmed by their clinical records from the Zhongshan Ophthalmic Center. We did not perform an in-scanner verification of uncorrected visual acuity immediately before or after the scan session, primarily to minimize participant burden during an already lengthy scanning protocol. Consequently, the exact degree of subjective blur may have varied slightly across individuals. However, all HM participants verbally confirmed that the stimuli appeared substantially blurred during the scan.

*Post hoc* sensitivity analyses were conducted using G*Power. With 24 participants in the HM group and 15 in the NV group, a two-tailed alpha of 0.05, and statistical power of 0.80, the present study had adequate sensitivity to detect a large effect of approximately Cohen’s d = 0.95. For an F test with one numerator degree of freedom, the minimum detectable effect was approximately Cohen’s *f* = 0.46, corresponding to a partial η^2^ of approximately 0.18. Thus, the current sample size was primarily adequate for detecting large effects, whereas statistical power to detect small-to-moderate effects was limited.

### 2.2 MRI acquisition

Blood oxygenation level-dependent (BOLD) signals were used as a measure of neural activation. Participants were scanned in a 3.0 Tesla Siemens MRI scanner at the MRI center of South China Normal University. Functional images were acquired using a T2*-weighted echo-planar imaging sequence with the following parameters: TR = 2000 ms, TE = 30 ms, FOV = 192 mm, flip angle FA = 90°, matrix size = 64 × 64, slice thickness = 3.0 mm (no gap). Each volume consisted of 32 axial slices covering the whole brain. A high-resolution T1-weighted anatomical image was also acquired for each participant for functional image registration and localization (TR = 2,300 ms, TE = 3.24 ms, FOV = 256 mm, FA = 9°, matrix size = 256 × 256, slice thickness = 1 mm, 176 sagittal slices).

### 2.3 Stimuli and experimental design

The stimulus presentation and experimental design were identical to those described in our previous study with NV participants (Cui et al., 2021). Briefly, participants were presented with two audiovisual movie segments (each 15 min long) using Psychtoolbox: a thriller clip (The Conjuring 2) and a scenic clip (Time Scapes). The thriller segment was specifically selected to capture the most emotionally intense and suspenseful “highlight” moments of the film (e.g., the climactic horror sequences involving the nun apparition and subsequent jump scares), which are known to reliably elicit strong threat-related responses. The scenic segment was chosen for its visually rich landscapes and emotionally neutral, calming content, serving as a contrast in both valence and arousal.

Critically, both clips contain no dialogue throughout the entire 15-min duration, which eliminates potential confounds related to language comprehension and semantic processing. This design choice ensured that the observed neural responses could be attributed primarily to the audiovisual emotional content and its interaction with visual blur, rather than to differential language processing between groups. Both clips have also been used in previous naturalistic fMRI studies (Cui et al., 2021). Participants viewed the stimuli via a mirror attached to the head coil and heard the soundtracks through MRI-compatible headphones. All participants confirmed they had not seen the clips before the experiment and were unaware of the study’s purpose.

Each participant completed two scanning sessions one week apart. Each session consisted of two 15-min runs, one for each movie. The order of movie presentation was counterbalanced across participants but remained consistent for each individual across the two sessions. This resulted in four conditions per participant: first viewing of the thriller (Thriller1), first viewing of the scenic movie (Scenic1), second viewing of the thriller (Thriller2), and second viewing of the scenic movie (Scenic2). The viewing procedure is illustrated in Figure 1.

**Figure 1:**
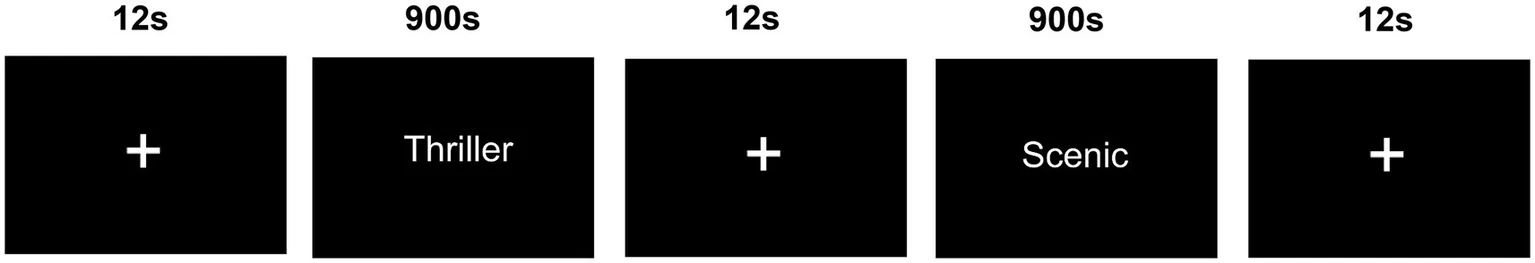
Each session began with a 12-s fixation period to allow for hemodynamic response stabilization. Participants then watched two 15-min movie clips (one thriller, one scenic) in a counterbalanced order. The session concluded with a 12-s fixation cue.

### 2.4 Data analysis

#### 2.4.1 Preprocessing

Functional MRI data were preprocessed using the AFNI software package (Cox, 1996). The first 12 s (6TRs) of each run were discarded to account for magnetic saturation and allow participants to adapt to the stimulus. Preprocessing steps included despiking, head motion correction, spatial normalization to the Talairach template (TT_N27), intensity scaling (to percent signal change from the mean baseline), removal of baseline drift, and spatial smoothing with a 4 mm full-width half-maximum Gaussian kernel. No participant exceeded 3 mm of maximum displacement, and no significant group differences in motion parameters were found. For each participant, there were four preprocessed functional time series of each voxel. The resulting data were used for subsequent ROI-based and whole-brain analyses.

#### 2.4.2 Regions of interest (ROIs)

Identical to our previous study, several ROIs known to be involved in sensory and emotional processing were defined anatomically based on Brodmann area templates (TT_N27 in AFNI) using Talairach coordinates. These included the amygdala, primary visual cortex (BA17), primary auditory cortex (BA41), anterior cingulate cortex (BA24), and dorsolateral prefrontal cortex (BA9).

#### 2.4.3 ROIs analysis

To examine the connectivity between ROIs and to distinguish consensual (group-level) from idiosyncratic (individual-level) responses, we extracted the time series of several ROIs (BA17, BA41, BA9, BA24) and correlated them with two amygdala reference signals for each participant:

(i) the leave-one-out group-averaged amygdala response, computed as the average of the amygdala time series across all other participants, which served as an index for the consensual (shared) component of the amygdala response; (ii) the participant’s own amygdala response, which served as an index for the idiosyncratic (individual) component.

All correlation coefficients were converted to Fisher’s z-values for subsequent statistical analysis. The same procedure was applied using the primary visual cortex (BA17) and the primary auditory cortex (BA41) as seed regions. This leave-one-out approach ensures that each participant’s own data are excluded from the group template, avoiding circularity between the consensual and idiosyncratic components.

#### 2.4.4 Amygdala-based functional connectivity analysis

The time series of activity was extracted from the amygdala ROI for each participant and each of the four movie-watching conditions. For each condition, whole-brain, voxel-wise functional connectivity analysis was performed using the amygdala time series as the seed (Li et al., 2025; Tagliazucchi et al., 2016). Correlation coefficients (r) between the amygdala seed and every other voxel in the brain were calculated and then converted to Fisher’s z-values for normality to facilitate group-level statistical comparisons. This analysis was used to examine condition-related patterns of amygdala-centered functional coupling across the whole brain.

#### 2.4.5 Intra-subject correlation analysis

To assess the similarity of brain activity between the first and second viewings of the same movie, intra-subject correlation analysis was performed. For each participant and each voxel, the Pearson correlation coefficient was calculated between the BOLD time series from the two viewings (e.g., Thriller1 vs. Thriller2). These correlation coefficients were also converted to Fisher’s z-values.

#### 2.4.6 Control analyses for low-level audiovisual features

To control for differences in low-level audiovisual physical properties between the two film clips, we separately extracted low-level visual and auditory features from each stimulus. Specifically, each video frame was first converted to grayscale. Mean grayscale intensity was calculated as a measure of luminance, whereas the standard deviation of grayscale intensity was used as a measure of contrast. Motion was quantified as the mean magnitude of optical flow estimated using the Horn–Schunck method, and spatial frequency was characterized by the mean high-frequency power derived from the two-dimensional Fourier power spectrum. These visual features were then averaged within each temporal window corresponding to one fMRI repetition time (TR = 2 s).

Auditory features were extracted from the audio tracks of the videos. Stereo audio was first converted to mono, after which three acoustic features were computed within each TR-length time window: root mean square (RMS) energy, spectral centroid, and spectral entropy. The resulting low-level visual and auditory features were then organized into regressors temporally aligned with the fMRI time series. In AFNI, each regressor was convolved with the GAM hemodynamic response function using waver. The seven convolved regressors were subsequently combined into a nuisance-regressor matrix. These low-level audiovisual nuisance regressors were removed from the BOLD time series using AFNI’s *3dTproject*. The residual BOLD time series were then used for the subsequent ISC and functional connectivity analyses.

#### 2.4.7 Statistical analysis

For the Intra-subject correlation and FC results obtained from the procedures described above, including FC estimates from both Run 1 and Run 2, independent-samples t-tests were conducted to compare neural responses between the HM and NV groups while viewing the same film. All statistical maps were converted to Z-statistic maps. Whole-brain analyses used a two-tailed voxel-wise threshold of *p* < 0.001 as the cluster-forming threshold, followed by cluster-size–based correction for multiple comparisons using AFNI’s *3dClustSim*. Cluster correction was performed using an NN = 1 neighborhood definition, with the cluster-level family-wise error (FWE) rate controlled at α = 0.05.

## 3 Results

### 3.1 Altered intra-subject synchrony and cross-modal compensation in HM

We first examined the similarity of neural responses within the HM group. Replicating the pattern observed in the NV group, intra-subject correlation analysis revealed that brain activity was significantly more similar between the two viewings for the thriller than for the scenic movie (*p* < 0.001), indicating greater temporal stability of neural responses during repeated viewing of emotionally arousing and suspenseful naturalistic stimuli.

However, direct comparisons between the HM and NV groups revealed critical differences. As illustrated in Figure 2, the NV group exhibited significantly higher intra-subject correlation than the HM group across both movie types, suggesting that degraded visual input in high myopia was associated with reduced neural response consistency across repeated viewings. Several ROIs remained significant after cluster-based correction (see Tables 1, 2). For the thriller, this difference was widespread, encompassing bilateral primary visual cortices (BA17), orbitofrontal cortex, precuneus, thalamus, right inferior frontal gyrus, and right insula. For the scenic movie, the regions with higher intra-subject correlation in the NV group were more limited.

**Figure 2:**
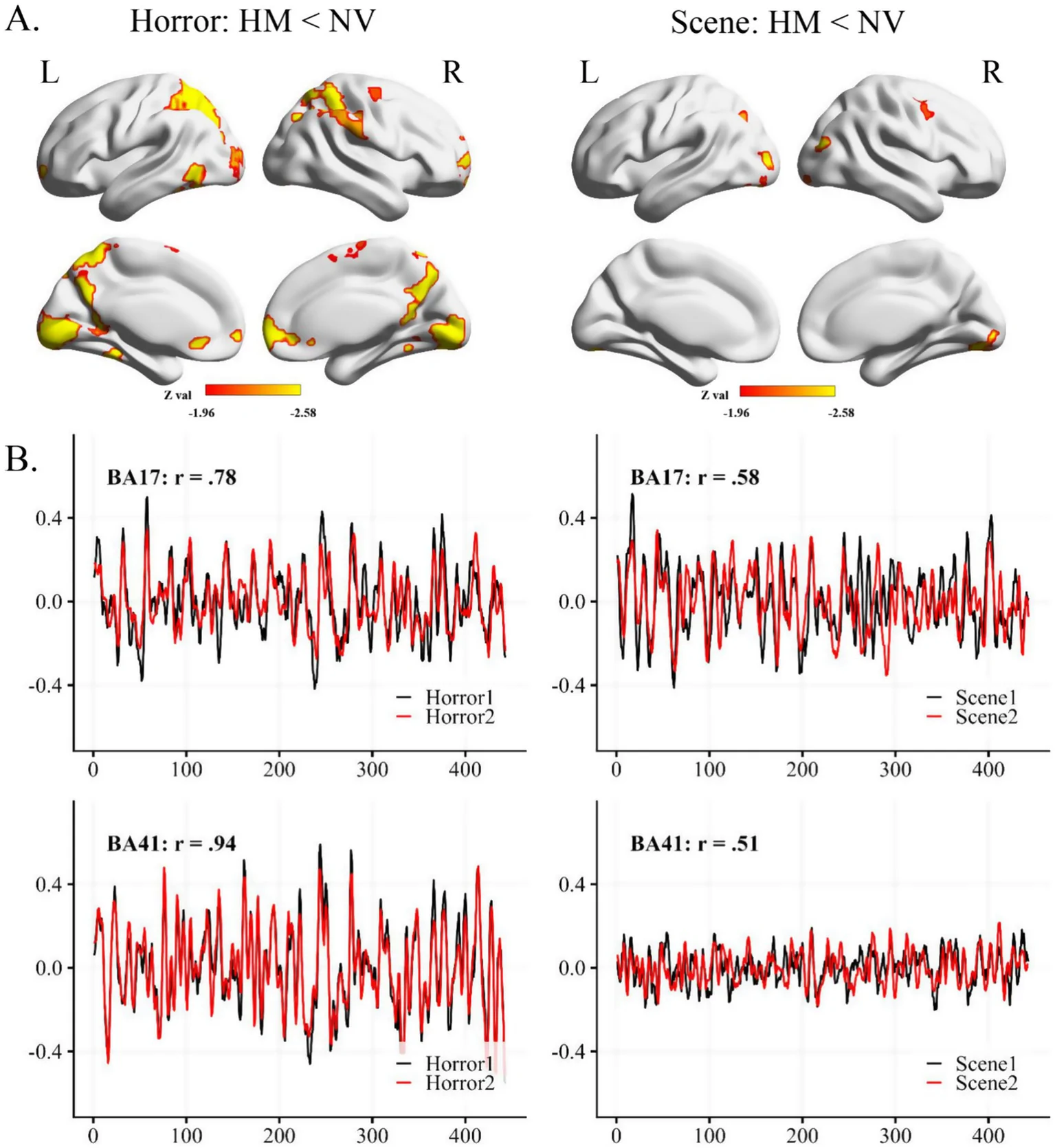
Altered intra-subject synchrony and cross-modal compensation in HM. **(A)** Brain regions (highlighted in red) where the NV group exhibited significantly higher intra-subject correlation than the HM group during repeated viewing of the thriller (left panel) and the scenic movie (right panel) (*p* < 0.001, cluster correction). **(B)** The MRI signal changes in two brain regions (i.e., BA17, BA41) revealed that repeated viewings of the horror movie elicited greater similarity in auditory cortex activity than in visual cortex, whereas no such differential pattern was observed with scenic movie.

**Table 1:** Effect sizes for cluster-based ROI t-tests: horror intra-subject correlation.

ROI	Voxels N	MNI x, y, z	Label in AAL3	Hedges g (95% CI)
1	3,778	(−5.1, −67.8, 20.5)	Calcarine_L	−1.89 (−2.65, −1.12)
2	617	(32.8, 62.2, −27.2)	OFCant_R*	−2.41 (−3.25, −1.58)
3	612	(−30.1, 61.1, −27.4)	OFCant_L*	−2.19 (−3.00, −1.39)
4	524	(5.8, 56.5, 0.0)	Frontal_Sup_Medial_R	−2.34 (−3.17, −1.52)
5	153	(58.6, −32.1, 37.5)	SupraMarginal_R	−1.71 (−2.45, −0.97)
6	129	(30.1, −13.0, 57.2)	Frontal_Sup_2_R	−1.70 (−2.44, −0.96)
7	120	(0.9, −3.0, 64.7)	Supp_Motor_Area_L	−1.56 (−2.29, −0.84)
8	83	(41.7, 24.2, 35.8)	Frontal_Mid_2_R	−1.70 (−2.44, −0.96)

^*^indicates that the centroid was located in an unlabeled background area of the AAL3 atlas (Rolls et al., 2020); the listed label corresponds to the nearest AAL3 region.

**Table 2:** Effect sizes for cluster-based ROI t-tests: scene intra-subject correlation.

ROI	Voxels N	MNI x, y, z	Label in AAL3	Hedges g (95% CI)
1	574	(4.4, −81.9, −6.3)	Lingual_R	−1.73 (−2.48, −0.99)
2	137	(−26.2, −63.2, 38.0)	Occipital_Mid_L	−1.60 (−2.33, −0.87)
3	119	(38.2, 0.0, 47.9)	Precentral_R	−1.94 (−2.71, −1.17)
4	103	(−25.8, 62.8, −30.2)	OFCant_L*	−1.69 (−2.43, −0.95)
5	82	(−39.9, −4.5, 49.5)	Precentral_L	−1.84 (−2.59, −1.08)
6	79	(−50.4, 14.4, 11.5)	Frontal_Inf_Oper_L	−1.70 (−2.44, −0.96)
7	77	(−28.3, −14.2, 11.4)	Putamen_L	−1.57 (−2.29, −0.84)

^*^indicates that the centroid was located in an unlabeled background area of the AAL3 atlas (Rolls et al., 2020).

Notably, while the HM group showed reduced synchrony in visual regions, they exhibited significantly enhanced synchrony in the primary auditory cortex (BA41) during movie viewing compared to the NV group, suggesting a cross-modal compensatory mechanism whereby auditory processing is enhanced when visual input is degraded. This pattern further implies that participants with high myopia may rely more strongly on auditory cues to maintain coherent perceptual and affective processing under conditions of reduced visual clarity.

### 3.2 Distinct functional connectivity patterns involving the amygdala and visual streams

We next investigated how chronic visual degradation alters functional connectivity patterns with the amygdala. A whole-brain, seed-based functional connectivity analysis was conducted using the amygdala as the seed region.

To examine how chronic visual degradation modulates amygdala-centered functional connectivity across different viewing conditions, we generated whole-brain, voxel-wise functional connectivity maps using the amygdala as the seed. This procedure was performed separately for each combination of the three factors: Group (HM vs. NV), Viewing times (first vs. second), and Movie type (thriller vs. scenic), resulting in a total of eight (2 × 2 × 2) whole-brain connectivity maps (one per condition).

A three-way ANOVA (factors: movie type, viewing time, group) performed on these functional connectivity results revealed a significant interaction effect, indicating that amygdala-centered functional coupling differed as a function of both stimulus context and visual status. Post-hoc tests indicated that compared to participants with NV, individuals with HM exhibit altered processing in both the ventral and dorsal streams when perceiving subjectively blurred visual stimuli. As illustrated in Figure 3, when participants were exposed to the horror movie for both viewings, the whole-brain functional connectivity network with the amygdala as the seed region showed significantly stronger connectivity between the dorsal stream and the amygdala in highly myopic participants than in those with NV. Enhanced connectivity was also observed among the auditory cortex, prefrontal cortex and the amygdala. Several ROIs remained significant after cluster-based correction (see Tables 3–6). These findings suggest that when the brain is unable to clearly discern potentially threatening visual information, it may adopt a “simplified, coarse, yet efficient” strategy to process such information. Due to degraded visual input, highly myopic individuals appear to employ compensatory mechanisms to maximize the efficiency of fear-related information processing and emotion regulation, thereby increasing functional integration between the auditory cortex, prefrontal cortex and the amygdala.

**Figure 3:**
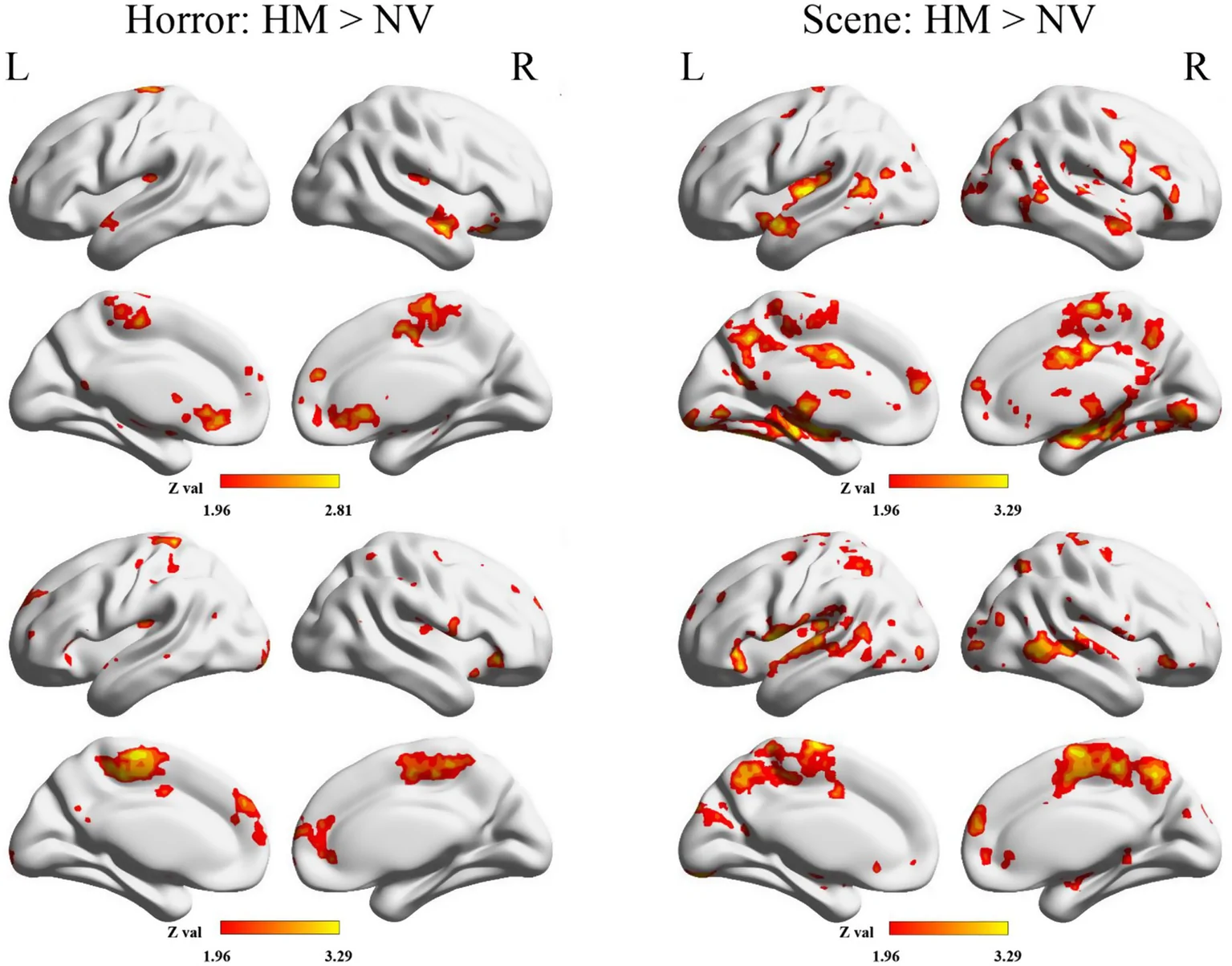
Strengthened amygdala-dorsal stream connectivity in HM during emotional movie viewing. Whole-brain functional connectivity maps with the amygdala as the seed region, contrasting the HM group versus the NV group. Results are displayed separately for the first viewing (upper panel) and the second viewing (lower panel). Warm colors (red) indicate brain regions where the HM group showed significantly stronger positive functional connectivity with the amygdala compared to the NV group (*p* < 0.001, cluster correction).

**Table 3:** Effect sizes for cluster-based ROI t-tests: run 1 horror FC.

Voxels N	MNI x, y, z	Label in AAL3	Hedges g (95% CI)
1,013	(52.8, 11.9, −23.0)	Temporal_Pole_Sup_R	2.50 (1.65, 3.35)
325	(−16.4, 6.3, −18.6)	Olfactory_L	2.09 (1.30, 2.88)
321	(−51.8, −6.6, −36.7)	Temporal_Inf_L	2.01 (1.23, 2.79)
147	(18.8, −2.8, −16.5)	Amygdala_R	2.17 (1.37, 2.98)
141	(−25.2, 63.9, −33.4)	OFCant_L*	−1.75 (−2.50, −1.00)
120	(−66.7, −54.9, −13.7)	Temporal_Mid_L*	1.64 (0.91, 2.38)
118	(−39.1, 42.2, −20.8)	OFClat_L*	1.61 (0.87, 2.34)
94	(60.7, −69.7, −12.6)	Temporal_Inf_R*	1.88 (1.12, 2.64)

^*^indicates that the centroid was located in an unlabeled background area of the AAL3 atlas (Rolls et al., 2020).

**Table 4:** Effect sizes for cluster-based ROI t-tests: run 1 scene FC.

ROI	Voxels N	MNI x, y, z	Label in AAL3	Hedges g (95% CI)
1	518	(0.9, −86.8, −32.8)	Cerebellum_Crus2_L	1.87 (1.10, 2.63)
2	515	(60.6, −10.2, −33.5)	Temporal_Inf_R	2.48 (1.63, 3.33)
3	298	(−54.0, −9.0, −34.2)	Temporal_Inf_L	1.72 (0.98, 2.46)
4	283	(−21.4, 65.1, −33.5)	OFCmed_L*	−2.22 (−3.02, −1.41)
5	165	(24.9, −102.2, −13.3)	Occipital_Inf_R	1.99 (1.22, 2.77)
6	152	(16.3, 27.1, −24.9)	OFCmed_R	2.07 (1.28, 2.86)

^*^indicates that the centroid was located in an unlabeled background area of the AAL3 atlas (Rolls et al., 2020).

**Table 5:** Effect sizes for cluster-based ROI t-tests: run 2 horror FC.

ROI	Voxels N	MNI x, y, z	Label in AAL3	Hedges g (95% CI)
1	820	(−47.1, −0.1, −36.9)	Temporal_Inf_L	2.43 (1.59, 3.27)
2	494	(41.3, 29.6, −19.2)	OFCpost_R	2.27 (1.46, 3.09)
3	466	(51.6, −1.8, −38.0)	Temporal_Inf_R	1.96 (1.19, 2.74)
4	433	(−55.3, 20.3, −8.8)	Frontal_Inf_Orb_2_L*	2.03 (1.24, 2.81)
5	409	(−66.2, −57.3, −1.8)	Temporal_Mid_L*	2.29 (1.47, 3.12)
6	386	(−11.6, 68.0, 38.7)	Frontal_Sup_2_L*	1.86 (1.10, 2.62)
7	304	(−32.8, −100.3, −7.5)	Occipital_Mid_L*	1.91 (1.14, 2.68)
8	287	(65.0, −63.2, −10.2)	Temporal_Inf_R*	2.12 (1.32, 2.91)
9	131	(−64.6, 68.5, 4.1)	Frontal_Mid_2_L*	−2.29 (−3.11, −1.47)
10	129	(−5.9, 71.6, −6.7)	Frontal_Med_Orb_L*	1.51 (0.79, 2.23)
11	124	(−69.5, −90.0, 0.9)	Occipital_Mid_L*	−2.23 (−3.05, −1.42)

^*^indicates that the centroid was located in an unlabeled background area of the AAL3 atlas (Rolls et al., 2020).

**Table 6:** Effect sizes for cluster-based ROI t-tests: run 2 scene FC.

ROI	Voxels N	MNI x, y, z	Label in AAL3	Hedges g (95% CI)
1	2,487	(−46.8, 13.4, −19.7)	Temporal_Pole_Sup_L	2.94 (2.02, 3.86)
2	953	(54.4, 25.0, −8.4)	Frontal_Inf_Orb_2_R	3.10 (2.15, 4.04)
3	683	(−53.6, −77.3, −2.5)	Occipital_Mid_L	2.76 (1.87, 3.65)
4	660	(55.7, −7.9, −36.7)	Temporal_Inf_R	2.19 (1.39, 3.00)
5	447	(51.1, −80.0, −5.9)	Occipital_Inf_R	2.76 (1.87, 3.65)
6	274	(9.7, 65.8, 68.0)	Frontal_Sup_Medial_R	−3.54 (−4.57, −2.52)
7	127	(−19.5, 69.6, 37.4)	Frontal_Sup_2_L*	1.78 (1.03, 2.53)

^*^indicates that the centroid was located in an unlabeled background area of the AAL3 atlas (Rolls et al., 2020).

For the scenic movie, a more complex pattern emerged. During the first viewing, the HM group exhibited stronger connectivity between the amygdala and both the dorsal and ventral visual streams (the latter being associated with detailed object recognition, or the “what” stream) compared to the NV group. However, by the second viewing, this pattern shifted: the enhanced connectivity with the dorsal stream remained significantly greater in the HM group, while the ventral stream connectivity reduced a lot significantly.

Control analyses using the parahippocampal place area (PPA) and primary sensory cortices (BA17, BA41) as seeds have not revealed these specific differences in visual stream engagement, confirming that the observed effects were specific to the emotional processing network centered on the amygdala.

### 3.3 Absence of consensual-idiosyncratic dissociation in HM

A critical finding from the NV group was the dissociation between brain networks correlating with consensual (group-average) versus idiosyncratic (individual-residual) amygdala responses upon repeated viewing. This dissociation was substantially reduced or absent in the HM group.

In the HM group, the functional connectivity patterns associated with both the consensual and idiosyncratic components of the amygdala response showed similar reductions upon the second viewing for both movie types. This suggests that under blurred viewing conditions, the clear separation between bottom-up, stimulus-driven consensual processing and top-down, experience-driven idiosyncratic processing is substantially reduced. The brain’s response appears to rely more uniformly on individual subjective experience to disambiguate the degraded visual input, thereby substantially reducing the neural dissociation seen in normally sighted individuals.

Control analyses using the parahippocampal place area (PPA) and primary sensory cortices (BA17, BA41) as seeds did not reveal these specific differences in visual stream engagement, confirming that the observed effects were specific to the emotional processing network centered on the amygdala.

## 4 Discussion

Our findings show that when individuals with HM view emotionally salient stimuli under blurred conditions, their brain responses differ in three main ways: cross-modal auditory enhancement, a dorsal stream bias, and an attenuation of canonical processing modes. These patterns likely reflect acute, state-dependent compensatory responses to degraded sensory input (Dong et al., 2025; Guo et al., 2010). To illustrate these findings, we propose a model (Figure 4) that contrasts the standard emotional processing circuit under clear viewing with the response patterns observed under blurred viewing.

**Figure 4:**
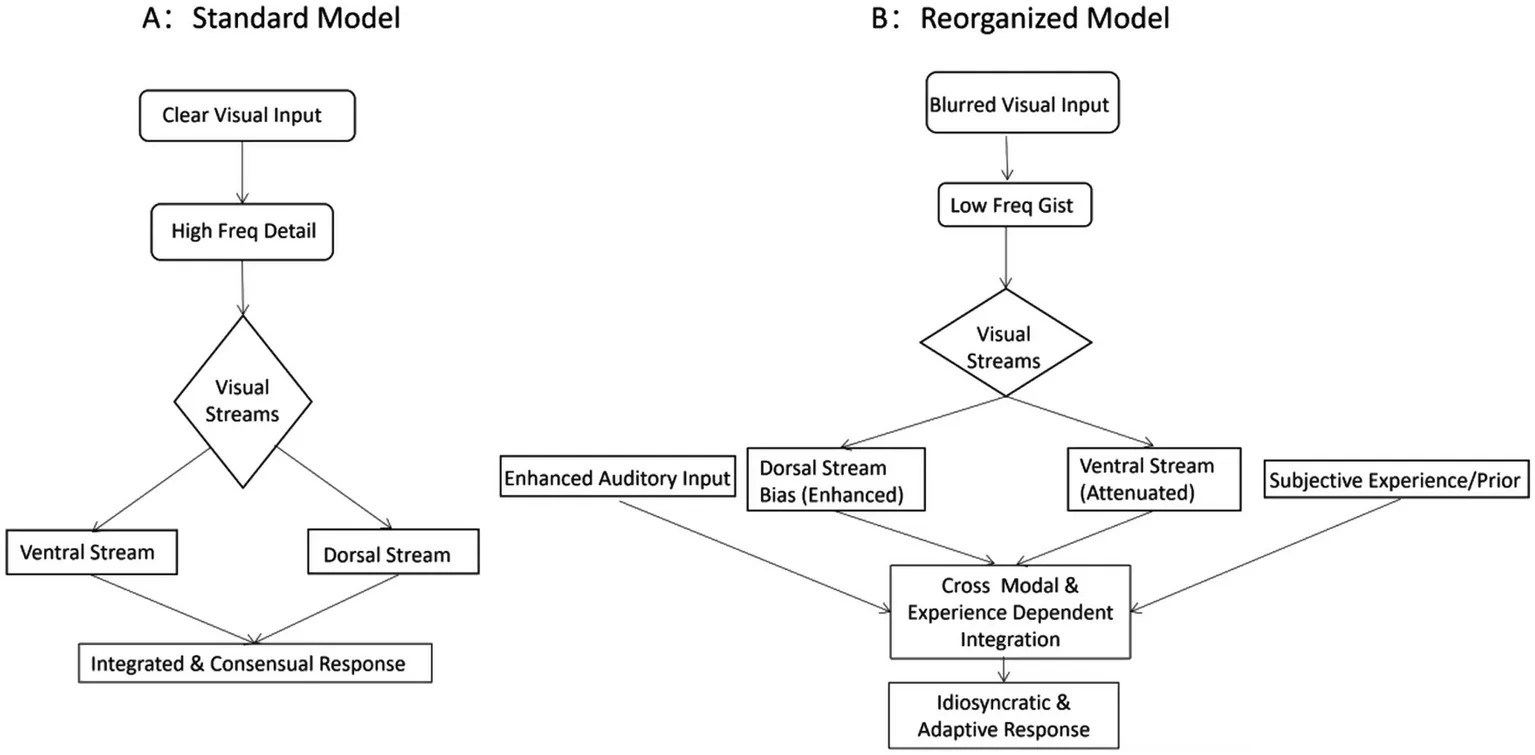
A schematic model of neural response patterns during emotional processing under blurred viewing conditions. **(A)** Standard model under clear viewing conditions. Clear visual input engages parallel processing via the ventral stream (for fine-grained object recognition) and the dorsal stream (for spatial/motion analysis), yielding a robust consensual, stimulus-driven response. **(B)** Observed response patterns under blurred viewing conditions (as experienced by HM participants). Degraded visual input is associated with a functional re-weighting of visual streams, resulting in a dorsal stream bias during threat processing. This is accompanied by cross-modal enhancement of auditory processing and increased reliance on top-down modulation from subjective prior experience. Together, these response patterns are associated with a reduced consensual-idiosyncratic dissociation relative to NV under clear viewing conditions.

Most notably, this model suggests three changes in HM:

1. Cross-modal auditory enhancement. When visual information is blurred, we observed enhanced intra-subject correlation in the auditory cortex during repeated viewing of the thriller movie. This pattern suggests that the brain may compensate for degraded visual input by increasing its reliance on intact auditory cues (Dong et al., 2025; Guo et al., 2010; Tian et al., 2024). Moreover, our findings extend the classic “dual visual streams” framework from visual neuroscience to emotional processing under degraded visual input.
2. Dorsal stream bias and state-dependent reweighting. In the HM group, emotional processing was associated with stronger functional connectivity between the amygdala and the dorsal visual stream. This suggests that when visual input is degraded, the brain may shift its processing toward the dorsal stream, which carries coarse, low-spatial-frequency information and is less affected by blur (Bar et al., 2006; De Gardelle and Kouider, 2010; Kveraga et al., 2007).
3. Reduced consensual–idiosyncratic dissociation. The reduced dissociation between consensual and idiosyncratic responses in the HM group may reflect a greater reliance on top-down, experience-based processing when bottom-up visual input is degraded (Cui et al., 2021; Engell et al., 2007). Under such conditions, shared, stimulus-driven responses across individuals may be weakened, while more personalized, experience-driven responses become relatively more prominent (Frith and Dolan, 1997; McMains and Kastner, 2011; Finn et al., 2020; Nastase et al., 2019; Nummenmaa et al., 2012). Consistent with this view, HM participants viewing blurred stimuli showed lower group-level synchronization, and the dissociation observed in NV under clear viewing conditions was substantially reduced (Barrett, 2017).

These findings may also be relevant to other populations with degraded vision, such as individuals with cataract, corneal opacities, or uncorrected refractive errors. The dorsal stream bias and cross-modal auditory enhancement observed here may reflect a more general compensatory response to blurred visual input (Bavelier and Neville, 2002; Stein and Stanford, 2008). From a rehabilitation perspective, training that emphasizes low-spatial-frequency motion cues may be more useful than detail-based training, and pairing blurred vision with sound may help support emotion recognition (Shams and Seitz, 2008; Stein and Stanford, 2008). At the same time, the greater reliance on top-down processing under blurred viewing conditions suggests that interventions should also consider potential maladaptive consequences, such as anxiety under sensory uncertainty (Grupe and Nitschke, 2013). We interpret these responses as potentially compensatory, as they may support relatively efficient threat detection when vision is degraded. However, without behavioral measures, we cannot determine whether they are behaviorally adaptive or maladaptive, and whether they extend to other emotions (e.g., happiness, disgust, sadness) remains an open question.

Several limitations should be considered when interpreting the present findings. A key methodological decision was to scan HM participants without optical correction, so that we could capture their subjective experience of visual blur. This approach allowed us to examine neural responses under ecologically valid blurred conditions, but it also means that acute, state-dependent responses to immediate blur cannot be fully separated from any long-term neuroadaptive changes associated with chronic myopia (Webster et al., 2002). Our findings should therefore be interpreted mainly as reflecting how the brain processes emotional information under blurred viewing conditions, rather than as definitive evidence of stable, trait-like neural changes. In addition, although all HM participants met the inclusion criterion of ≤ −6.00 D based on clinical records and confirmed that the stimuli appeared substantially blurred, we did not perform in-scanner verification of uncorrected visual acuity before or after scanning. The exact degree of subjective blur may therefore have varied slightly across individuals. Future studies should examine whether the observed effects scale with myopia severity by correlating individual SER values with effect sizes (e.g., BA41 Intra-subject Correlation).

We also did not collect behavioral measures, such as threat detection accuracy, reaction times, or emotional intensity ratings. Although we interpret the observed neural response patterns as potentially compensatory, we cannot directly link them to behavioral outcomes or adaptive function. In addition, the cross-sectional design identifies associations between HM and altered neural responses, but it cannot establish causality. The generalizability of our findings is also limited by the sample and the stimuli. Our sample size (*N* = 39) is comparable to or larger than many previous neuroimaging studies using naturalistic paradigms in clinical populations, and a *post hoc* sensitivity analysis (see Methods 2.1) indicated that the present sample was mainly adequate for detecting large effects. However, the relatively modest sample size still limits the precision of effect size estimates (Button et al., 2013; Turner et al., 2018). A lack of significant dissociation in a modest sample should not be taken as evidence that the mechanism is biologically absent. Finally, the sample consisted mainly of young adults, and only two movie clips (one thriller and one scenic) were used for each emotional category. These factors limit how far our findings can be generalized to other age groups or to a broader range of emotional contexts (Westfall et al., 2015). The observed effects may also be specific to the particular editing style of The Conjuring 2 and Time Scapes and may not fully generalize to other threat or calm contexts.

Several directions for future work remain. The most important next step would be to test NV participants viewing the same movies with artificial blur, and to scan HM participants while wearing their optical correction. Such a design would help separate the immediate effects of degraded sensory input from any long-term neuroadaptive changes associated with chronic visual impairment in HM. Beyond functional measures, diffusion tensor imaging (DTI) (Basser et al., 1994) could be used to examine whether structural changes in white matter tracts connecting visual, auditory, and limbic regions underlie the functional response patterns we observed. It would also be valuable to link these neural patterns to behavioral measures, such as threat detection accuracy and reaction times in ecological tasks, which would help connect neural responses to real-world emotional perception and behavior.

In conclusion, this study identifies a set of functional response patterns—cross-modal auditory enhancement, a dorsal stream bias, and an attenuation of canonical processing modes—that characterize emotional processing in individuals with HM viewing blurred stimuli. These findings suggest that classic models of threat perception, which are based on clear vision, may not fully capture neural responses when vision is degraded. Our findings provide a preliminary framework for understanding state-dependent neural responses under sensory constraint, and may be useful for future studies of emotion network function in other populations with visual degradation.

## Data Availability

The raw data supporting the conclusions of this article will be made available by the authors, without undue reservation.
